# Exploring biomarkers of premature ovarian insufficiency based on oxford nanopore transcriptional profile and machine learning

**DOI:** 10.1038/s41598-023-38754-x

**Published:** 2023-07-17

**Authors:** Zhaoyang Yu, Mujun Li, Weilong Peng

**Affiliations:** 1 The First Affiliated Clinical College of Guangxi Medical University, Nanning, China; 2 Reproductive Medicine Research Center, The First Affiliated Hospital of Guangxi Medical University, Nanning, China; 3 School of Computer Science and Cyber Engineering, Guangzhou University, Guangzhou, China

**Keywords:** Biotechnology, Computational biology and bioinformatics, Genetics, Biomarkers, Medical research

## Abstract

Premature ovarian insufficiency (POI) is a reproductive endocrine disorder characterized by infertility and perimenopausal syndrome, with a highly heterogeneous genetic etiology and its mechanism is not fully understood. Therefore, we utilized Oxford Nanopore Technology (ONT) for the first time to characterize the full-length transcript profile, and revealed biomarkers, pathway and molecular mechanisms for POI by bioinformatics analysis and machine learning. Ultimately, we identified 272 differentially expressed genes, 858 core genes, and 25 hub genes by analysis of differential expression, gene set enrichment, and protein–protein interactions. Seven candidate genes were identified based on the intersection features of the random forest and Boruta algorithm. qRT-PCR results indicated that *COX5A*, *UQCRFS1*, *LCK*, *RPS2* and *EIF5A* exhibited consistent expression trends with sequencing data and have potential as biomarkers. Additionally, GSEA analysis revealed that the pathophysiology of POI is closely associated with inhibition of the PI3K-AKT pathway, oxidative phosphorylation and DNA damage repair, as well as activation of inflammatory and apoptotic pathways. Furthermore, we emphasize that downregulation of respiratory chain enzyme complex subunits and inhibition of oxidative phosphorylation pathways play crucial roles in the pathophysiology of POI. In conclusion, our utilization of long-read sequencing has refined the annotation information within the POI transcriptional profile. This valuable data provides novel insights for further exploration into molecular regulatory networks and potential biomarkers associated with POI.

## Introduction

Premature ovarian insufficiency (POI) is a reproductive endocrine syndrome characterized by hypergonadotropic hypoestrogenic amenorrhea in women aged < 40 years^1^. Approximately 1% and 0.01% of women aged 40 and 20 years, respectively, are diagnosed with POI^2^. The etiology of POI is heterogeneous and ranges from cases of spontaneous or idiopathic development to those resulting from specific causes including chromosomal abnormalities, autoimmune diseases, infections, and iatrogenic factors (namely surgery, radiation, and chemotherapy)^3^. However, the etiology of POI in most cases remains unknown, and genetic factors are considered the most important contributors to its progression^4^. POI causes infertility due to the destruction of reproductive potential and is associated with a high risk of long-term complications, including cardiovascular diseases, genitourinary symptoms, osteoporosis, and neurological impairment^5^. Therefore, it is essential to understand the regulatory networks controlled by specific genes to determine the pathogenesis and therapeutic targets of POI.

High-throughput sequencing technology is a powerful tool for interpreting the human genetic code. However, first-generation sequencing (Sanger) and next-generation sequencing (NGS) fail to accurately decipher complete transcript information and structural variations due to read-length limitations and incomplete assembly. The third-generation sequencing technology represented by Oxford Nanopore Technology (ONT) overcomes these limitations. Its principle is to identify the type of base by real-time detection of electrical signals from nanopores^6,7^. The advantage of ONT is that it produces ultra-long reads with an average length of over 10 kb and improves the quality of genomic assembly^8^, thereby solving long and complex repetitive regions in various species^9^. From the advent of the first portable sequencer, MinION, to the latest PromethION platforms, ONT sequencing has been widely used in medicine and has achieved remarkable results.

Machine learning (ML) has become an important tool in medicine in recent years. Various classical algorithms of ML such as random forest (RF), extreme gradient enhancement (XGBoost), support vector machine (SVM), and the latest popular Boruta algorithm, make important contributions to predictive models of diseases, prognostic models, and marker identification^10,11^. ML excels at solving complex, interactive, and nonlinear relationships, and improves the quality of data analysis through processes such as feature screening and dimensionality reduction^12^. In this study, we characterize the full-length transcript profile for the first time based on ONT sequencing, and revealed the biomarkers, pathway and molecular mechanisms for POI by bioinformatics analysis and machine learning.

## Materials and methods

### Collection of samples and clinical data

Five POI and five control women matched for age and body mass index (BMI) were included in the study. The diagnostic criteria for POI were as follows^1^: (i) age < 40 years; (ii) at least 4 months of oligo/amenorrhea; and (iii) serum basal follicle-stimulating hormone (FSH) > 25 IU/ml was detected on two occasions in > 4 weeks interval. The exclusion criteria were as follows: (i) diagnosis of other endocrine disease, (ii) history of ovarian surgery, and (iii) 3 months of hormones use before blood collection. The control group included infertile women who visited the hospital due to tubal factors, with normal menstrual cycles and basic sex hormones. Common exclusion criteria included a history of oral hormone therapy within the past three months, other endocrine or serious systemic diseases, as well as previous pelvic or ovarian surgery. All participants underwent a 12-h fasting period and provided 2.5 ml of peripheral blood using a PAXgene Blood RNA tube (BD, United States) on days 2–4 of the menstrual cycle. Clinical data including anti-Mullerian hormone (AMH), FSH, luteinizing hormone (LH), estradiol (E2), progesterone (P), testosterone (T), prolactin (PRL), antral follicle count (AFC), age, and BMI were collected. All participants signed an informed consent form. This study was approved by the Ethics Committee of the First Affiliated Hospital of Guangxi Medical University (NO. 2021KY-E-249) and conformed to the guidelines and regulations stated in the Declaration of Helsinki.

### cDNA library construction and full-length transcript identification

Total RNA was extracted using a matching kit (PAXgene Blood Kit, BD, America), a cDNA library was constructed for qualified samples (RNA concentration > 40 ng/µL, OD260/280 ratio between 1.7 and 2.5, RIN value ≥ 7). Transcriptome sequencing was performed using the PromethION platform (Oxford Nanopore Technologies, Oxford, UK). The full-length sequence was polished to obtain a consensus sequence, and then compared with the human reference genome by Minimap2 software. The sequence with identity < 0.9 and coverage < 0.85 was filtered, and the final de-redundant sequence was used in subsequent analysis.

### Analysis of differentially expressed transcripts and genes

The expression levels of transcripts or genes were measured as counts per million (CPM). The CPM is calculated as follows: CPM = R/T × 1,000,000; where, “R” represents the number of reads aligned to a particular transcript and “T” indicates the total number of fragments aligned to the reference transcriptome. The DESeq2 R package was used for differential expression analysis of full-length transcripts of the 10 samples. Differentially expressed transcripts (DETs) and genes (DEGs) were screened based on the criteria of fold change (FC) > 1.5 and false discovery rate (FDR) < 0.05, with FDR values obtained through adjustment of raw *P* values using the Benjamini–Hochberg method.

### Functional annotation and enrichment analysis of DEGs

DEGs were aligned to the following database: Gene Ontology (GO, http://www.geneontology.org), Kyoto Encyclopedia of Genes and Genomes (KEGG, http://www.genome.jp/kegg/). Comprehensive information for functional annotation and enrichment analysis was obtained using BLAST.

### Gene set enrichment analysis (GSEA)

GSEA was performed using the C2.KEGG gene set and Hallmark gene set as reference gene sets for alignment with transcriptional expression profiles. Enrichment score (ES) is a statistic that evaluates the degree of gene enrichment. The gene sets were normalized based on their size to obtain a normalized enrichment score (NES), which reflects the degree of gene enrichment. NES > 0 suggests that gene sets were enriched at the top of the list, indicating pathways activated in the POI group. Conversely, NES < 0 suggests that gene sets were enriched at the bottom of the list, indicating pathways inhibited in the POI group. |NES|> 1 and *P* < 0.05 was defined as a significantly enriched pathway. The core genes were found to be the major contributors to the enrichment score^13^.

### Identification of hub genes by PPI

We further identified the differentially expressed core genes by the intersection of DEGs and core genes from KEGG and Hallmark dataset. The intersecting genes were uploaded to the STRING database to construct PPI networks and imported into Cytoscape software for visualization. Respectively, the top 15 hub genes from each differential core gene set were identified using the clustering coefficient algorithm in the CytoHubba plugin, and finally a total of 30 hub genes were obtained. The differential core genes after removing duplicates were the primary focus of our study.

### Filtering key features based on the RF and Boruta algorithms

**Figure 1:**
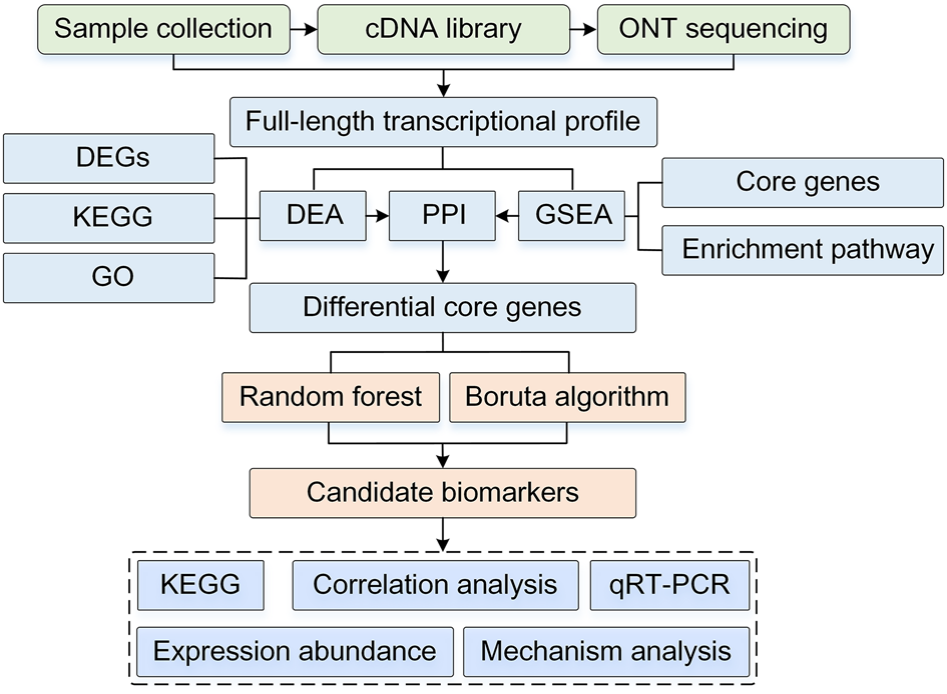
Flow charts based on analysis of bioinformatics and machine learning. DEA: differential expression analysis, DEGs: differentially expressed genes, GSEA: gene set enrichment analysis, PPI: protein–protein interactions.

Random Forest (RF) is an integrated tree-based machine learning classification tool that combines the idea of adaptive nearest neighbor with bagging. RF detects correlations and interactions between variables through the grouping property of trees and uses variable importance to select and rank variables^14^. Boruta is a feature selection method for supervised classification^15^. Z-values for each attribute are obtained at each iteration, and shaded Z-values are generated by random shuffling of the true features. A true feature is considered to be significant if its Z-value is greater than the maximum Z-value of the shaded feature over multiple independent trials. We identified reliable biomarkers by the intersection of two machine learning algorithms. The analysis process based on bioinformatics and machine learning was shown in Fig. 1.

### Quantitative real-time PCR (qRT-PCR) assay

We recollected 20 POI and 20 control peripheral blood samples for qRT-PCR assay. Monocytes were extracted using lymphocyte isolation liquid (Solefault), and total RNA was extracted from monocytes using TRizol reagent (Invitrogen). First-strand cDNA was obtained using the reverse transcription tool of SweScript All-in-One First-Strand cDNA (Servicebio). qRT-PCR was performed with cDNA and SYBR Green qPCR Master Mix (ServiceBio). Each step was performed according to the manufacturer's instructions. Candidate genes were normalized by *GAPDH*, and expression levels were calculated using the 2-ΔΔCt method. The primers for seven mRNAs were summarized in Supplementary Table S1.

### Statistical analysis

SPSS 24.0 was used for clinical characteristic analysis, and GraphPad Prism 6.0 was used for candidate gene data analysis and visualization. Normality and variance tests were conducted before statistical analysis. Continuous variables with normal or nearly normal distribution were analyzed using Student's t-test and expressed as mean ± SD, while Non-normally distributed data were analyzed using Mann–Whitney U test and expressed as median (quartile). Pearson correlation coefficient was used for correlation analysis. RF and Boruta analyses were performed with Python sklearn version 0.22.1 and R language Boruta version 8.0.0, respectively. Fisher's Exact Test determined the significance level of enrichment pathways with *P* < 0.05 considered statistically significant.

### Ethical approval

The study was approved by the Ethics Committee of the First Affiliated Hospital of Guangxi Medical University. All participants gave written informed consent.

## Results

### Clinical characteristics of participants

The clinical characteristics of two groups are shown in Supplementary Table S2. Patients in the POI group had significantly lower level of AMH and higher level of FSH and LH than those in the control group (*P* < 0.05). There was no significant difference in age, BMI, E2, P, PRL, and T between the two groups (*P* > 0.05). Notably, there was no significant reduction in the E2 level in the POI group. We speculated that premature follicular development in the early POI stage leads to a temporary increase in estrogen levels, while follicular depletion in the late POI stage often leads to a significant decline in E2 levels.

### Overview of ONT transcriptome sequencing

The clean data output of each sample ≥ 3.77 GB. The N50 and average read length of ranged from 782–1,016 bp and 877–1,071 bp, respectively. The maximum read length was 189,497 bp. The length distribution of reads ranged from 1 kb to > 10 kb, with 1 kb long reads accounting for the majority. The average quality value was between Q11 to Q12. The proportion of full-length transcripts was 90.2–92.84% (Supplementary Table S3).

### Expression and identification of DETs and DEGs

**Figure 2:**
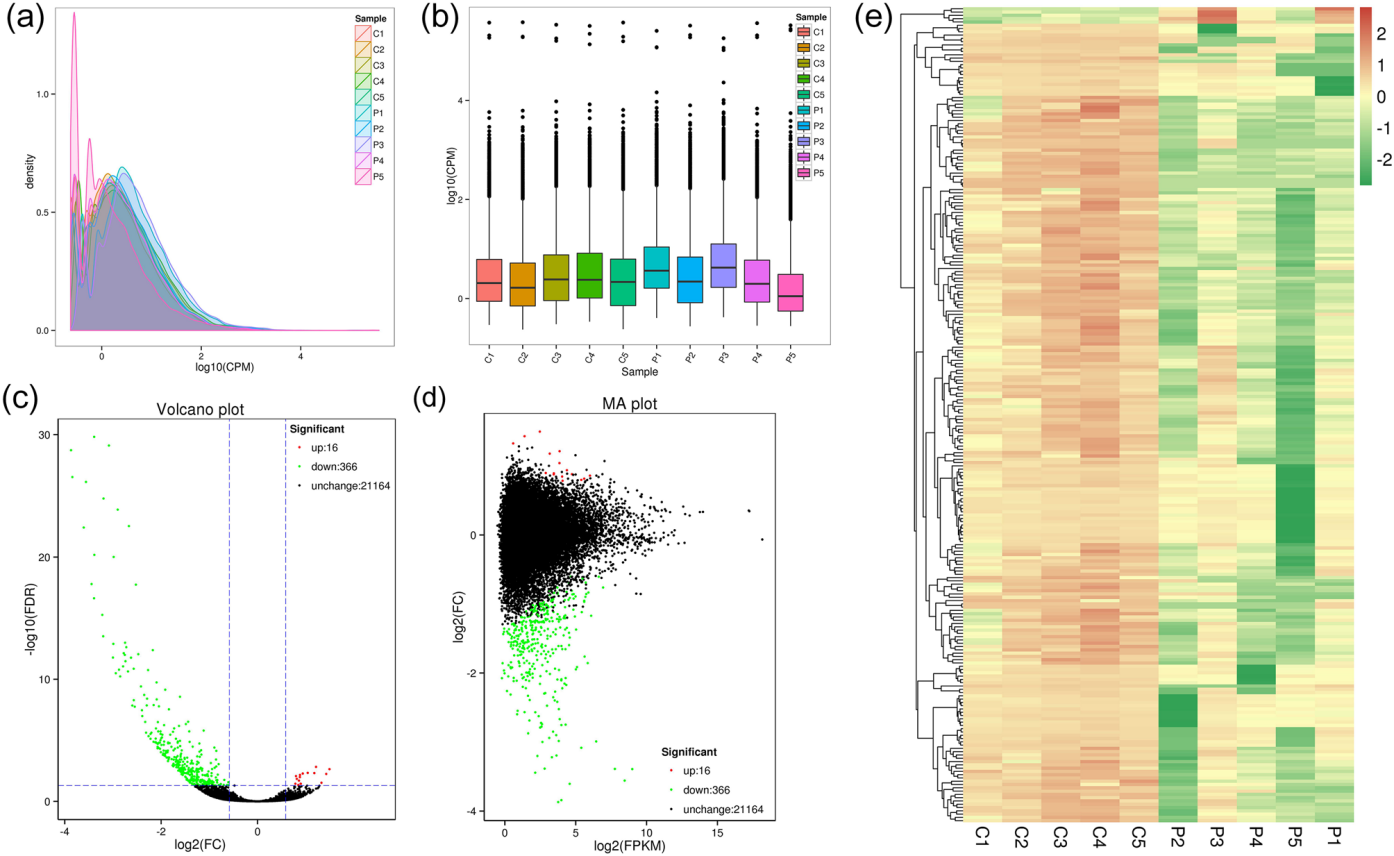
Overall transcript expression levels and differentially expressed transcripts (DETs) in the two groups. (**a**) CPM density distribution map. (**b**) CPM boxplot of each sample. (**c**) Volcano plot of DETs. (**d**) MA plot of DETs. (**e**) Cluster heatmap of DETs expression.

We showed the expression level of 10 samples by density distribution plot and boxplot of CPM (Fig. 2a, b). Ultimately, 26,122 transcripts were identified after full-length sequence redundancy, with 13,593 new transcripts and 7,724 novel gene loci identified after comparison with the reference genome. In addition, 382 DETs (366 downregulated and 16 upregulated transcripts in the POI group) and 272 DEGs (255 downregulated and 17 upregulated genes in the POI group) were identified by differential expression analysis (Supplementary Table S4). Volcano and MA plots depicted the differences in transcript expression levels and fold change between the two groups, and clustering heat maps showed the expression patterns of the DETs (Fig. 2c–e).

### Functional enrichment analysis of DEGs

Functional annotation classification of the GO database suggested that cellular processes, single-organism processes, metabolic processes, reproduction, and reproductive processes were highly expressed in biological processes. Cells, cell parts, organelles were enriched in cellular components. Additionally, binding, catalytic activity, and molecular transducer activity were highly expressed items in molecular functions (Supplementary Fig.S1a). KEGG pathway enrichment analysis revealed that the DEGs were significantly enriched in five major pathways (Supplementary Fig.S1b). The cellular processes included endocytosis, cellular senescence, and oocyte meiosis. The major enriched entries of the environmental information process were the PI3K-Akt, mTOR, and nuclear transcription factor kappa B (NF-κB) signaling pathways. Genetic information processing included the protein processing pathway in the endoplasmic reticulum and RNA transport. The human diseases included amyotrophic lateral sclerosis, fluid shear stress, atherosclerosis, and Alzheimer's disease. Finally, osteoclast differentiation, platelet activation, and T-cell receptor signaling pathways were the most enriched items in organismal systems.

### GSEA and identification of core genes

**Figure 3:**
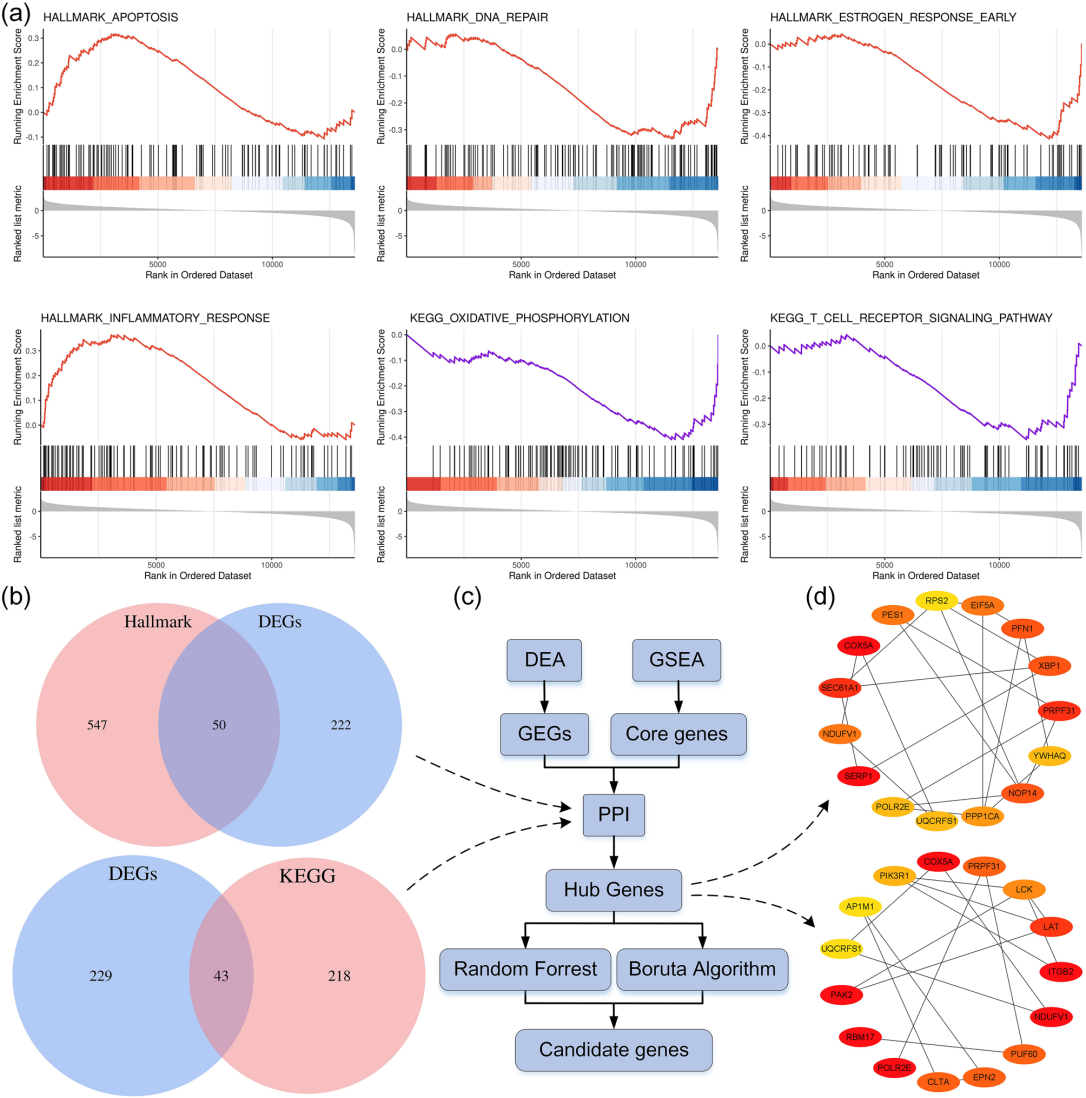
Identification of differential core genes. (**a**) GSEA. (**b**) Venn diagram of DEGs and core genes. (**c**) Flow chart for identification of candidate genes. (**d**) Identification of hub genes by PPI.

By aligning with the hallmark gene set, 17 significantly enriched pathways (|NES|> 1, *P* < 0.05) and 597 core genes were identified, including oxidative phosphorylation pathway (NES = -1.86, *P* = 0.000), late estrogen response (NES =  − 1.78, *P* = 0.000), NF-κB pathway (NES = 1.69, *P* = 0.000), inflammatory response (NES = 1.69, *P* = 0.000), response to unfolded proteins (NES =  − 1.71, *P* = 0.0024), early estrogen response (NES =  − 1.60, *P* = 0.031), PI3K-AKT signalling pathway (NES =  − 1.60, *P* = 0.008), apoptosis (NES = 1.41, *P* = 0.015), glycolysis (NES =  − 1.44, *P* = 0.025), DNA damage repair (NES =  − 1.37, *P* = 0.041). By comparison with the KEGG gene set, a total of 11 significantly enriched pathways and 261 core genes were obtained (|NES|> 1, *P* < 0.05), including lysosomal (NES =  − 1.73, *P* = 0.001), oxidative phosphorylation (NES =  − 1.64, *P* = 0.003), Alzheimer's disease (NES =  − 1.56, *P* = 0.005), Huntington's disease (NES =  − 1.51, *P* = 0.006), endocytosis (NES =  − 1.52, *P* = 0.007) , ribosomes (NES =  − 1.43, *P* = 0.03), natural killer cell-mediated cytotoxicity (NES =  − 1.37, *P* = 0.04), T cell receptor signalling pathway (NES =  − 1.38, *P* = 0.048), etc. (Fig. 3a). The detailed information of GSEA is shown in Supplementary Table S5.

### Identification of hub genes using PPI

50 and 43 differential core genes were identified by the intersection of two core gene sets and DEGs (Fig. 3b, Supplementary Table S6). The results were uploaded to the STRING database for PPI analysis and further imported into Cytoscape for visualization. The top 15 hub genes were screened based on clustering coefficients. Finally, a total of 25 hub genes were determined after merging and de-duplication: *SERP1, UQCRFS1, LCK, PES1, ITGB2, RPS2, PPP1CA, YWHAQ, PFN1, NOP14, POLR2E, XBP1, NDUFV1, SEC61A1, EIF5A, LAT, PIK3R1, RBM17, COX5A, PAK2, PUF60, PRPF31, EPN2, CLTA, and AP1M1* (Fig. 3c, d).

### Identification of feature variables based on machine learning

**Figure 4:**
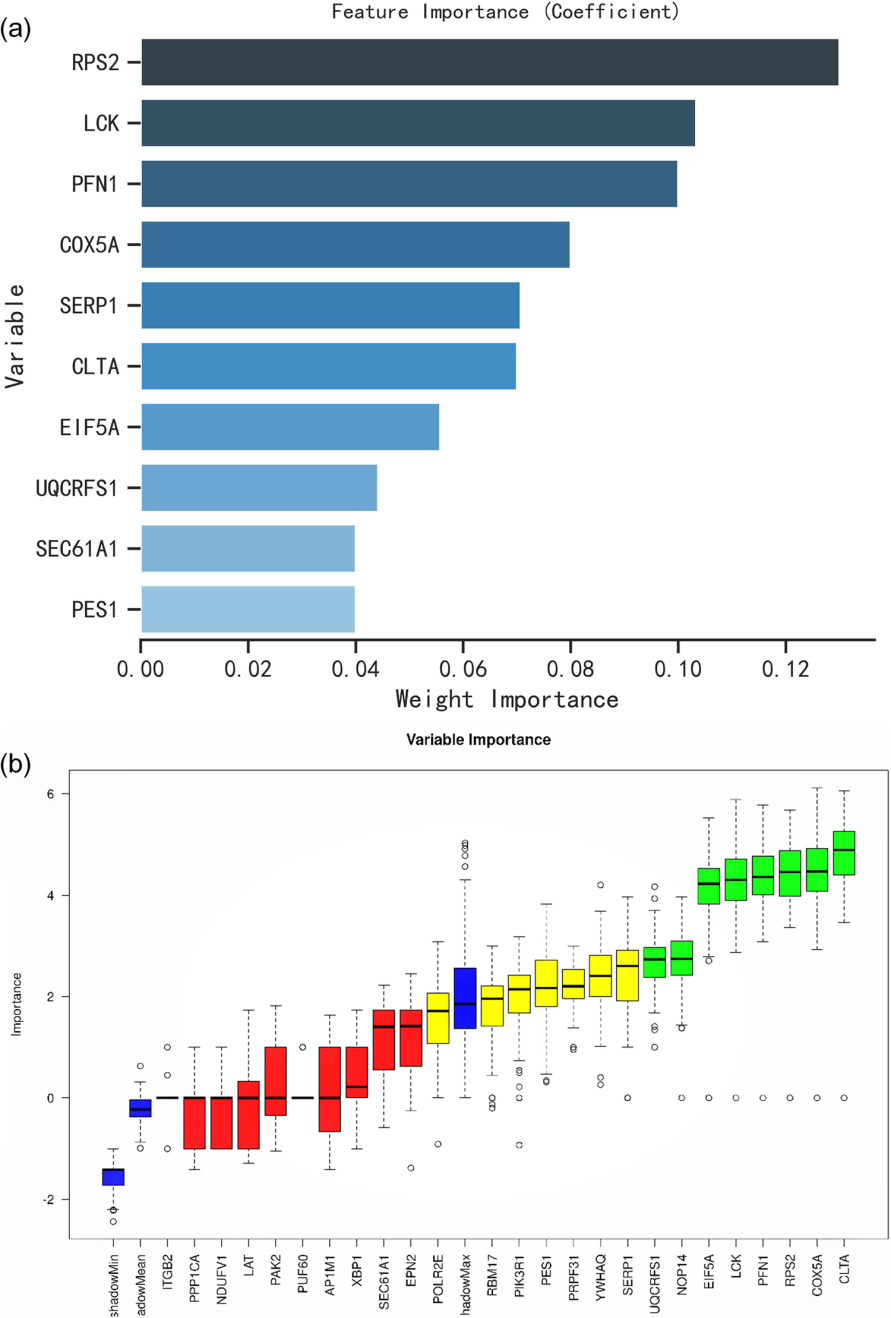
Screening of candidate biomarkers based on machine learning. (**a**) Top10 feature variables based on random forest. (**b**) Variable attribute classification based on Boruta algorithm. Green, red and yellow represented accepted, rejected and tentative attributes respectively.

Based on the impact factor importance analysis of RF, the top 10 feature variables were identified as follows: *RPS2*, *LCK, PFN1, COX5A, SERP1, CLTA, EIF5A, UQCRFS1, SEC61A1, NOP14,* and *PES1* (Fig. 4a). Based on the Boruta algorithm, eight important features with confirmation properties were screened as follows: *UQCRFS1, LCK, RPS2, PFN1, NOP14,*

*EIF5A, COX5A, and CLTA* (Fig. 4b). Two machine learning algorithms determined seven intersecting variables: *COX5A, UQCRFS1, LCK, RPS2, PFN1, EIF5A,* and *CLTA*, which were considered candidate biomarkers of important significance for the pathogenesis of POI.

### Relevance, expression and enrichment analysis of hub genes

**Figure 5:**
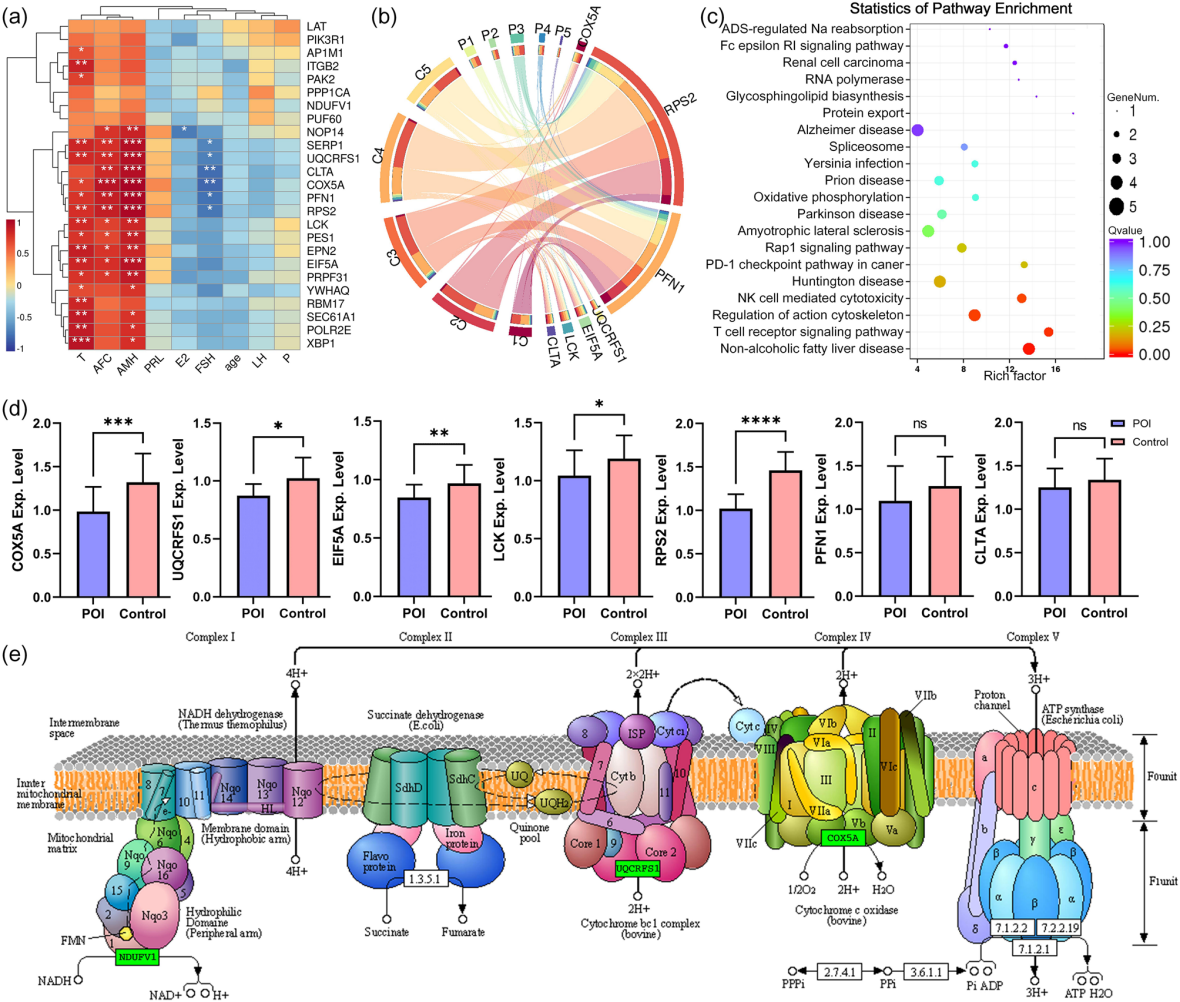
Relevance, expression and enrichment analysis of Hub genes. **(a**) Clinical Correlation Heat Map, * suggested *P* < 0.05, ** suggested *P* < 0.01. (**b**) Circos map of candidate gene expression. (**c**) Functional enrichment analysis of KEGG for hub gene. (**d**) qRT-PCR detection of candidate genes. (**e**) The down-regulated core genes, namely *NDUFV1*, *UQCRFS1* and *COX5A*, are subunits of mitochondrial respiratory chain complex I, complex III and complex IV^16^.

Correlations between the 25 hub genes and clinical indicators were demonstrated by correlation heat maps (Fig. 5a). Overall, hub genes were significantly positively correlated with AMH, AFC, and T (r > 0, *P* < 0.05) and negatively correlated with FSH (r < 0, *P* < 0.05). Circos plots demonstrate the abundance of biomarkers in the different samples. The results indicated that the expression of candidate biomarkers was significantly lower in the POI group than in the control group (Fig. 5b). KEGG enrichment analysis showed that the hub genes were enriched in pathways such as natural killer cell mediated, cytotoxicity regulation of actin cytoskeleton, T cell receptor signaling pathway, non-alcoholic fatty liver disease (Fig. 5c).

### Expression validation of candidate biomarkers using qRT-PCR

The expression of these candidate biomarkers were verified using qRT-PCR in an expanded sample size cohort. The results suggested that the level of *COX5A, UQCRFS1*, *LCK, RPS2,* and *EIF5A* were significantly downregulated in the POI group compared to the control group, which was consistent with the expression trend of ONT sequencing. In contrast, *CLTA* and *PFN1* were not statistically significantly different between the two groups (Fig. 5d). Therefore, our results indicate that *COX5A, UQCRFS1, LCK, RPS2, EIF5A* may be diagnostic biomarkers for POI.

## Discussion

This study is the first to characterize the transcriptional profile of POI using third-generation ONT sequencing through the state-of-the-art PromethION platform. Overall, 382 DETs and 272 DEGs were identified using differential expression analysis. KEGG enrichment analysis revealed that DEGs were mainly enriched in signaling pathways including PI3K-AKT, NF-κB, apoptosis, cellular senescence, and oocyte meiosis. Several studies have demonstrated that PI3K-Akt is an essential signaling pathway regulating primordial follicle recruitment, oocyte growth, and granulosa cell proliferation and differentiation^17,18^. PI3K/Akt pathway alterations can cause primordial follicle hyperactivation and granulosa cell apoptosis, leading to premature ovarian failure (POF) ^17^. The NF-κB is a key regulator of inflammation, aging, apoptosis, and immunity that promotes POI progression by regulating several pro-inflammatory factors such as tumor necrosis factor-α and interleukin 1^19^.

GSEA targets the entire gene expression profile without imposing a threshold, enabling identification of biologically significant genes that may not be significantly differentially expressed. Notably, in this study, pathways related to ovarian and mitochondrial function were predominantly suppressed in the POI group (NES < 0), including the PI3K/AKT/mTOR pathway, early and late estrogen response pathways, oxidative phosphorylation, and DNA damage repair. In contrast, the NF-κB pathway, inflammatory response, and apoptosis were activated in POI (NES > 0), which have been shown to induce granulosa cell apoptosis and ovarian dysfunction. Notably, GSEA analysis using both Hallmark and KEGG gene sets indicated inhibition of the oxidative phosphorylation pathway (NES < 0, *P* < 0.05). Oxidative phosphorylation is the primary mechanism by which the mitochondrial respiratory chain drives ADP to produce ATP. The inhibition of oxidative phosphorylation suggests that POI pathogenesis may be linked to mitochondrial dysfunction and impaired energy metabolism. Studies have shown that inhibiting oxidative phosphorylation or mitochondrial function can result in oocyte maturation arrest or apoptosis^20^. Recent studies have shown that inhibition of *SIRT1* expression by oxidative stress leads to impaired mitochondrial oxidative phosphorylation, causing follicular apoptosis and POI^20^.

The main function of mitochondria is to produce ATP by oxidative phosphorylation, a process transduced by four enzyme complexes (I to IV) and ATP synthase (complex V) in the mitochondrial respiratory chain (Fig. 5e)^16,21^. The main respiratory chain (NADH respiratory chain) is composed of complexes I, III, and IV, which cooperate to transfer electrons to molecular oxygen and generate an electrochemical gradient on the inner membrane to drive ATP production^22^. Electrons pass through these three complexes in turn to produce 90% of the ATP required to maintain cell life activities^23^. Mitochondrial dysfunction is a typical feature of tissue aging^24^. In our study, the candidate biomarkers *COX5A* and *UQCRFS1*, which encode subunits of mitochondrial respiratory chain complex III and IV respectively, were found to be significantly reduced in POI. The downregulation of these subunits may play a crucial role in the mechanism underlying oocyte senescence^25^.

Cytochrome C oxidase subunit Va (*COX5A*) is one of the subunits of cytochrome C oxidase (complex IV) in the mitochondrial respiratory chain. It constitutes the catalytic center of the enzyme and is believed to play an important role in regulating age-related oxidative phosphorylation^26^. Previous studies have shown that the deletion of *COX5A* leads to mitochondrial dysfunction in mouse embryos, resulting in apoptosis and reduced cell numbers in blastoderm embryos^27^. Recent studies have shown that *COX5A* expression is reduced in the hippocampus of aged mice and plays a critical role in aging-related cognitive degeneration, implying that *COX5A* may be a marker of aging or a potential target for anti-aging drugs^28^. The latest research suggests that inhibiting *CLPP* can reduce the content and activity of respiratory chain complex IV by affecting *COX5A*, leading to mitochondrial dysfunction and apoptosis in human ovarian granulosa cells. This indicates the expression and regulatory role of *COX5A* in ovarian function^29^. Similarly, Mitochondrial cytochrome C oxidase subunit II (*COX2*) and cytochrome C oxidase subunit III (*COX3*) were also reported to be significantly downregulated in GV oocytes in aging mice^25^.

Rieske iron-sulfur polypeptide 1 (*UQCRFS1*), one of the key subunits of panthenol-cytochrome c reductase, has catalytic and electron transfer properties and is involved in the final step of mitochondrial respiratory chain complex III assembly and is essential for enzymatic activity^21^. *UQCRFS1* has been shown to be dysregulated in prefrontal cortical degeneration, musculoskeletal dysfunction and in Alzheimer's disease^30^, which are commonly characterized by age-related aging disorders. Therefore, we hypothesize that *UQCRFS1* plays an important role in the regulation of aging mechanisms. Interestingly, we also unexpectedly found that the expression of the core gene *NDUFV1*, a subunit of ubiquinone oxidoreductase (complex I), was downregulated in POI. Complex I, as the main entrance to the respiratory chain, is the largest protein complex in the mitochondrial respiratory chain^31^, and its dysfunction is associated with mitochondrial disease, Parkinson's disease, and aging^31^. Mitochondria-encoded complex I have been proven to have age-related downregulation in mouse oocytes^25^. *NDUFV1* may be involved in the pathology of cognitive impairment in neurodegenerative disorders^32^. In summery, we found that expression of *NDUFV1*, *UQCRFS1*, and *COX5A*, the subunits of complexes (I, III and IV), are significantly downregulated in POI. This implies that POI patients present with mitochondrial dysfunction and impaired energy metabolism. Thus, we speculated that the downregulation of subunits of the respiratory chain enzyme complex and inhibition of the oxidative phosphorylation pathway play an important role in the pathogenesis of POI.

Eukaryotic translation initiation factor 5A (*EIF5A*) is thought to be an anti-aging factor whose expression level decreases in senescent cells. The deletion of *EIF5A* decreases ATP production and mitochondrial metabolic enzyme levels, and alters mitochondrial dynamics^33^. *EIF5A* mediates autophagy regulatory mechanisms at the translational level to reverse immune senescence in humans suggesting that *EIF5A* activation has potential for the treatment of senescence or age-related diseases^34^. Previous studies have demonstrated the beneficial role of *EIF5A* in mouse embryonic development and cell differentiation, and inhibition of its expression leads to abnormal NK cell function and increased embryonic loss^35^. Lymphocyte specific kinase (*LCK*) is a tyrosine kinase of the Src family that is widely expressed in various tissues and cells. Relevant studies have shown that *LCK* is a key mediator in the aging process^36^, but the conclusions are inconsistent. Early studies have shown a significant decrease in phosphorylated *LCK* in the lipid rafts of peripheral blood T lymphocytes in elderly subjects. This finding suggests that *LCK* plays an important role in age-related decline in T cell function^37^. However, recent studies have revealed that increased *LCK* expression and hyperleptinemia interact to induce inflammation and accelerate renal ageing^36^. Another study on reproductive aging in male mice shows that *LCK* level increases with age and is a key molecule in the aging of the cephalic end of the male epididymis^38^. This is contrary to our findings. It is speculated that there may be differences in the expression of *LCK* in male and female reproductive aging. Ribosomal proteins (RP) contribute to a range of reproductive processes, including oogenesis, spermatogenesis and embryogenesis^39–41^. Stage-like arrest of follicle growth was observed when *RPS2* expression was disturbed in female Culex paleus, supporting the possibility that the shutdown of RPS2 expression contributes to the arrest of ovarian development^40^. Through RNA interference with the expression of *RPL11* and *RPS2*, 42 and 30% of Phytoseiulus Persimilis individuals, respectively, do not lay eggs or hatch, while the remaining females experience shortened oviposition periods, reduced egg production, and reduced egg hatchability^42^. These evidences suggest that ribosomal proteins are closely related to reproduction. Inhibition of *RPS2* leads to arrest of follicular and ovarian development, which is consistent with the down-regulation of *RPS2* expression in POI. However, the mechanism of *RPS2* in human reproduction is currently lacking.

Although our research has shed new light on the biomarkers and pathogenesis of POI, there are certain limitations that need to be acknowledged. Firstly, we only conducted full-length transcriptome sequencing on peripheral blood samples. To validate the expression patterns and associations of markers across different sample types, further analysis is required on follicular fluid, granulosa cells, and ovarian tissue from animal models with POI. Furthermore, ovarian dysfunction is a progressive process that begins with the initial decline in ovarian reserve function and progresses to POI, ultimately culminating in POF. Therefore, relying solely on the transcriptional expression profile of POI may not accurately reflect the dynamic development of this disease process. Future analyses should be conducted at different stages to explore changes in transcriptional profiles and markers over time. Finally, The sample size in our study was limited. In the future, it is recommended to conduct multi-center cohort studies with larger samples to enhance the reliability of biomarkers.

## Conclusion

In conclusion, our study refined the transcriptional profile of POI through third-generation ONT sequencing. Seven candidate biomarkers were identified through bioinformatics and machine learning. GSEA revealed that inhibition of the PI3K-AKT pathway, oxidative phosphorylation, and DNA damage repair, as well as activation of inflammatory and apoptotic pathways, may be closely associated with the pathophysiology of POI. We have placed particular emphasis on the downregulation of enzyme complex subunits and inhibition of oxidative phosphorylation pathways, which are crucial in the pathogenesis of POI due to their ability to trigger mitochondrial dysfunction and impair energy metabolism. These findings shed new light on the investigation of molecular mechanisms of POI at the transcriptional level.

### Supplementary Information

Supplementary Figure S1.

Supplementary Table S1.

Supplementary Table S2.

Supplementary Table S3.

Supplementary Table S4.

Supplementary Table S5.

Supplementary Table S6.

## Data Availability

The raw RNA sequencing dataset can be accessed at NCBI under bioproject (accession number: PRJNA964483), which will become publicly available on September 1, 2023.
