# Author Correction: Exploring biomarkers of premature ovarian insufficiency based on oxford nanopore transcriptional profile and machine learning

**DOI:** 10.1038/s41598-023-40482-1

**Published:** 2023-08-16

**Authors:** Zhaoyang Yu, Mujun Li, Weilong Peng

**Affiliations:** 1 The First Affiliated Clinical College of Guangxi Medical University, Nanning, China; 2 Reproductive Medicine Research Center, The First Affiliated Hospital of Guangxi Medical University, Nanning, China; 3 School of Computer Science and Cyber Engineering, Guangzhou University, Guangzhou, China

Correction to: *Scientific Reports* 10.1038/s41598-023-38754-x, published online 17 July 2023

The original version of this Article contained an error in the order of the author names, which was incorrectly given as Zhaoyang Yu, Weilong Peng & Mujun Li. The correct order is Zhaoyang Yu, Mujun Li & Weilong Peng.

Additionally, an incorrect email address for author Mujun Li was quoted. Correspondence and requests for materials should be addressed to lmj0081@163.com

The original Article has been corrected.

