# Supplementary material for: Exploring biomarkers of premature ovarian insufficiency based on oxford nanopore transcriptional profile and machine learning
Source: Sci Rep. 2023 Jul 17;13:11498. doi: 10.1038/s41598-023-38754-x (PMC10352282; doi:10.1038/s41598-023-38754-x)
Supplement: Supplementary file 1 — Supplementary Figure S1. [file 41598_2023_38754_MOESM1_ESM.pdf]

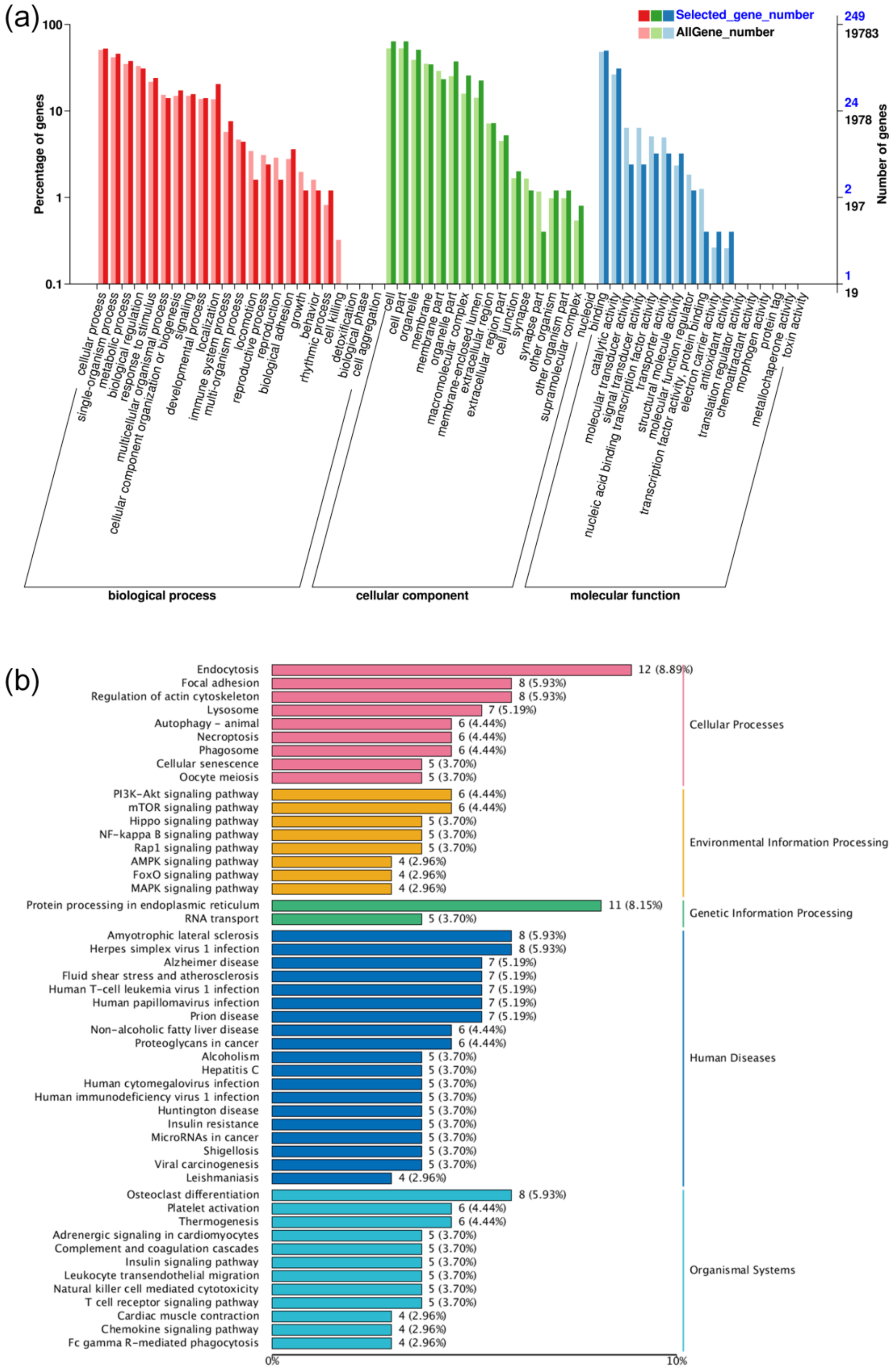

Supplementary Fig.S1 Functional annotation of DEGs in the GO and KEGG databases. (a) Functional classification of DEGs in GO. (b) Functional classification of DEGs in KEGG.
