# Supplementary material for: Exploring biomarkers of premature ovarian insufficiency based on oxford nanopore transcriptional profile and machine learning
Source: Sci Rep. 2023 Jul 17;13:11498. doi: 10.1038/s41598-023-38754-x (PMC10352282; doi:10.1038/s41598-023-38754-x)
Supplement: Supplementary file 2 — Supplementary Table S1. [file 41598_2023_38754_MOESM2_ESM.docx]

**Supplementary Table S1 Primer information of candidate genes**

| **Gene** | **Forward Primer** | **Reverse Primer** |
| --- | --- | --- |
| UQCRFS1 | TGCTTCTGCTGATGTGTTGGC | GCAAGGGCAGTAATAACCACCA |
| EIF5A  RPS2  PFN1  COX5A | CGTAAGAATGGCTTTGTGGTG  CTCCCTGCCTATTAAGGAATCAG  GTGGAACGCCTACATCGACAA  GTCACAGGAGACAGATGAGGAGTTT | TCCCAGTAAAGATGTCAATACCAAC  CACCTCCTTGGAGCACTTAACAC  TCTTTGCCAACCAGGACACC  AAAGTTGGTCTAAGTTCCTGGATGA |
| CLTA CGCAGCAAGAGAGCGAGATT  LCK TCTGCACAGCTATGAGCCCT | | CCATTTACGGATACTTTCAGGCT  GAAGGAGCCGTGAGTGTTCC |
