# Supplementary material for: Exploring biomarkers of premature ovarian insufficiency based on oxford nanopore transcriptional profile and machine learning
Source: Sci Rep. 2023 Jul 17;13:11498. doi: 10.1038/s41598-023-38754-x (PMC10352282; doi:10.1038/s41598-023-38754-x)
Supplement: Supplementary file 3 — Supplementary Table S2. [file 41598_2023_38754_MOESM3_ESM.docx]

**Supplementary Table S2 Clinical Characteristics of Included Participants**

| **Parameters** | **Control（n=5）** | **POI（n=5）** | ***P*-value** |
| --- | --- | --- | --- |
| Age (year) | 33.60±4.72 | 34.20±4.82 | 0.847 |
| BMI (kg/m^2^） | 21.05±1.81 | 20.94±1.42 | 0.92 |
| AMH (ng/mL) | 3.18±1.08 | 0.10±0.08 | 0.003** |
| FSH (mIU/mL) | 6.12±0.88 | 35.38±11.30 | 0.004** |
| LH (mIU/mL) | 4.99±1.38 | 18.46±9.70 | 0.015* |
| E2 (pg/L) | 30.54±7.52 | 52.56±34.02 | 0.224 |
| P(nmol/L) | 0.14±0.06 | 0.17±0.13 | 0.609 |
| T(nmol/L) | 0.30±0.15 | 0.17±0.10 | 0.152 |
| PRL (nmol/L) | 25.10±11.97 | 16.89±9.92 | 0.272 |
| AFC (n) | 11.80±2.77 | 2.20±1.48 | 0.000** |

[Abbreviation](javascript:;): BMI: body mass index, AMH: anti-Mullerian hormone, FSH: follicle-stimulating hormone, LH: luteinizing hormone, E2: estradiol, P: progesterone, T: testosterone, PRL: prolactin, AFC: antral follicle count.

* suggested P<0.05, ** suggested P<0.01.
